# Childhood adherence to a potentially healthy and sustainable Nordic diet and later overweight: The Norwegian Mother, Father and Child Cohort Study (MoBa)

**DOI:** 10.1111/mcn.13101

**Published:** 2020-10-25

**Authors:** Neha Agnihotri, Nina Cecilie Øverby, Elling Bere, Andrew Keith Wills, Anne Lise Brantsæter, Elisabet Rudjord Hillesund

**Affiliations:** ^1^ Department of Nutrition and Public Health University of Agder Kristiansand Norway; ^2^ Department of Health and Inequalities and Centre for Evaluation of Public Health Measures Norwegian Institute of Public Health Oslo Norway; ^3^ Department of Sport Science and Physical Education University of Agder Kristiansand Norway; ^4^ Faculty of Health Sciences University of Bristol Bristol UK; ^5^ Division of Infection Control and Environmental Health Norwegian Institute of Public Health Oslo Norway

**Keywords:** barker hypothesis, birth cohort, child nutrition, childhood obesity, dietary patterns, MoBa MBRN

## Abstract

The New Nordic Diet (NND) is a potentially healthy and sustainable dietary pattern represented by locally available and traditionally consumed foods in the Northern countries. The diet has been commonly examined in adult populations, but less is known regarding its potential associations with overweight/obesity in children. We have previously developed child diet scores measuring compliance to the NND at child age 6 and 18 months and 3 and 7 years. In this study, we aimed to describe child and maternal characteristics and assess potential associations between the age‐specific diet scores and child overweight at 8 years. This study is based on the Norwegian Mother, Father and Child Cohort Study (MoBa), including 14,989 mother–child pairs and uses data from the Medical Birth Registry of Norway (MBRN). The scores measured NND compliance as a total score and categorized into low, medium and high NND compliance at each age point. Using logistic regression models, we investigated the association between each age‐specific score and the odds of overweight at 8 years. In crude analyses, adherence to the NND at 6 months was inversely associated with odds of overweight at 8 years in the continuous score (odds ratio = 0.95, 95% CI [0.91, 0.98]) and when comparing high versus low NND adherence (odds ratio = 0.81, 95% CI [0.70, 0.94]). The association was almost entirely attenuated in the adjusted models. In conclusion, child NND adherence up to 7 years of age was not associated with odds of overweight at 8 years in adjusted analyses.

**Key messages**

- Dietary patterns established early in life may have an impact on later risk of childhood overweight/obesity.
- The New Nordic Diet is a potentially healthy and sustainable dietary pattern with foods from the Nordic countries. Little is known regarding compliance to the diet in early childhood and its association with later weight status in children.
- We could not demonstrate evidence for the New Nordic Diet to be protective against overweight at 8 years in our study.
- Future studies should assess adiposity with objective measurements.

## 1 INTRODUCTION

Between 1990 and 2016, the worldwide prevalence of overweight/obesity among children under 5 years increased from 32 to 41 million (World Health Organization, 2019). In Norway, more than 20% of the 8‐year‐olds were found to be overweight in the Norwegian Child Growth Study (Glavin et al., 2014). Despite an observed plateauing in some developed countries, overweight/obesity still remains one of the major public health challenges worldwide (Olds et al., 2011; Wabitsch, Moss, & Kromeyer‐Hauschild, 2014), particularly as the prevalence of childhood obesity in developing countries continues to increase (Bauman, Rutter, & Baur, 2019). Early prevention is important as overweight/obesity in childhood and adolescence may track into adulthood and is associated with adverse health, such as premature mortality, diabetes, cardiovascular disease and asthma (Reilly & Kelly, 2010). Furthermore, the first years of life have been increasingly acknowledged as a crucial period for overweight/obesity prevention (Baidal et al., 2016; Pietrobelli & Agosti, 2017), and there is a growing evidence suggesting that dietary patterns laid early in life may shape later eating preferences and track into and beyond childhood (Lioret et al., 2015; Mikkilä, Räsänen, Raitakari, Pietinen, & Viikari, 2005). Despite this, dietary patterns in relation to health outcomes are less commonly explored in children, compared with adult populations. In a systematic review from 2014, 80 diet quality indices that had been used to investigate health‐related outcomes among children and adolescents were reviewed (Marshall, Burrows, & Collins, 2014). The authors found that the most studied outcome was weight status, however, the findings were generally inconsistent.

In the mentioned review, none of the dietary indices assessing child diet were from Nordic countries, which signifies a gap in the current literature. The New Nordic Diet (NND) has been suggested as a regionally appropriate diet in the Nordic countries. It encompasses a concept of a potentially health‐promoting and sustainable diet with foods that carry a Nordic identity and are locally available in the Northern countries such as oats, rye, cabbages, root vegetables, apple, pears, berries and fish (Bere & Brug, 2009; Mithril et al., 2012; Mithril et al., 2013).

The Nordic dietary pattern has been examined in various versions, albeit mostly in adult populations. Recent studies in adults have shown the diet to be associated with improved health (Adamsson et al., 2011; Adamsson, Cederholm, Vessby, & Risérus, 2014; Poulsen et al., 2014) and weight (Hillesund, Bere, Haugen, & Øverby, 2014; Poulsen et al., 2014; Poulsen, Crone, Astrup, & Larsen, 2015; Skreden et al., 2018) and lower total mortality (Olsen et al., 2011). In children, the effects of serving school meals based on the NND to children aged 8–11 years were investigated in the Danish OPUS School Meal Study, and the intervention lasting over 6 months showed an improved dietary and nutritional quality of the consumed food compared to the control period (Andersen et al., 2014) and improved metabolic markers due to the increased fish intake (Damsgaard et al., 2016). However, the school meals also led to an increased waist circumference, which was positively associated with potato consumption, but not with android/total fat mass. Despite numerous beneficial findings in adults, the evidence regarding child adherence to the NND and associated health outcomes remains inconclusive. For instance, child adherence to the Mediterranean diet has been shown to be inversely associated with childhood obesity in children aged 2–9 years in eight European countries (Tognon et al., 2014). Less is known regarding adherence to the NND from an early age and potential associations with overweight/obesity in observational studies.

We have previously developed age‐specific child diet scores at age 6 and 18 months and 3 and 7 years, aiming to reflect NND compliance among children (Agnihotri et al., 2020). The scores were based on the rationale of the maternal NND score in The Norwegian Mother, Father and Child Cohort Study (MoBa; Hillesund et al., 2014). In the current study, the aims were (i) to describe child and maternal characteristics according to degree of compliance to the NND at 7 years and (ii) to investigate potential associations between the age‐specific child NND scores and odds of being overweight at 8 years.

## 2 MATERIALS AND METHODS

### 2.1 Study design and sample

The data used in this study derive from MoBa which is a prospective, population‐based pregnancy cohort study conducted by the Norwegian Institute of Public Health (Magnus et al., 2016). Pregnant women were recruited from all over Norway from 1999 to 2008. The women consented to participation in 40.6% of the pregnancies. The cohort now includes 114,500 children, 95,200 mothers and 75,200 fathers. The current study is based on version 8 of the quality‐assured data files released for research in February 2014. Follow‐up of the participants has been conducted through questionnaires at a regular interval and data collection is still on‐going. For the present analyses, data from seven questionnaires from MoBa were used: Q1 (at baseline, week 13–20 of the pregnancy), Q2 (week 22), Q4 (child age 6 months), Q5 (child age 18 months), Q6 (child age 3 years), Q8 (child age 7 years) and Q9 (child age 8 years). The dataset was linked to relevant data from the Medical Birth Registry of Norway (MBRN), which is a national health registry containing information about all births in Norway. Data collection of the 8‐year‐olds were still not complete in version 8 of the file. In total, 19,946 (46.1%) mothers had responded to Q9 when the data file was released.

**FIGURE 1:**
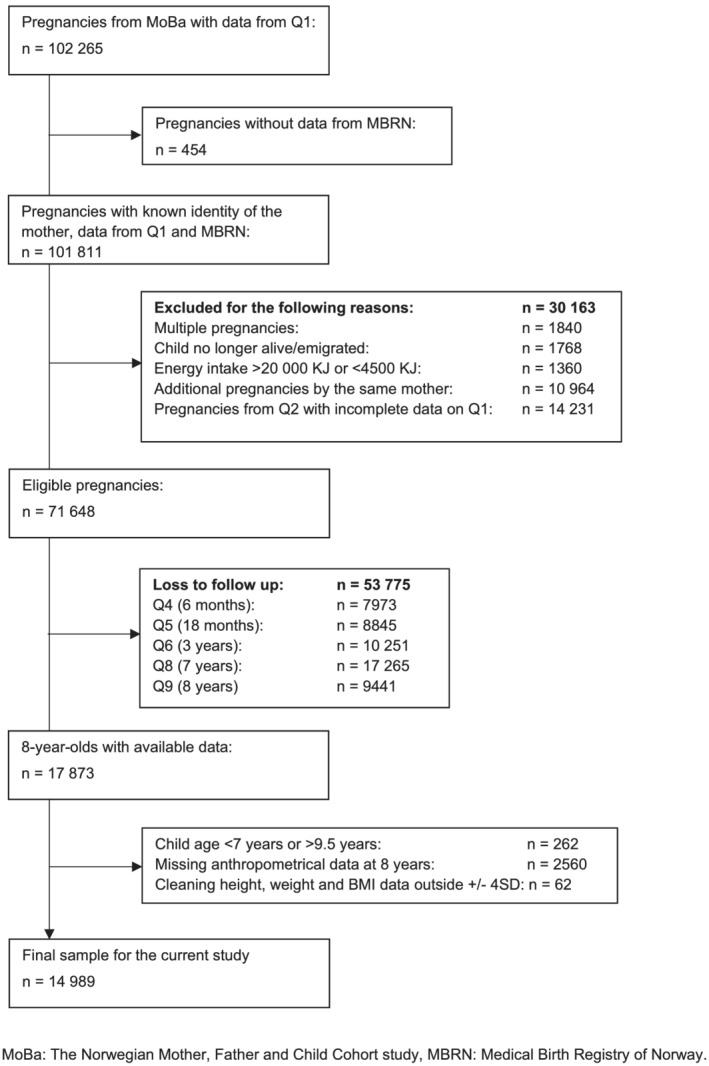
Flow chart of sample selection from the Norwegian Mother, Father and Child Cohort study

To be eligible for inclusion in the present study, MoBa participants had to have answered the baseline questionnaire (Q1) and be registered in the MBRN (*n* = 101,811). Figure 1 shows a flow chart of the sample selection.

We excluded multiple pregnancies (*n* = 1840), pregnancies where the child was no longer alive, or where the parents had emigrated (*n* = 1768), pregnancies with no dietary data (*n* = 14,231) and pregnancies with an implausible energy intake defined as <4,500 KJ or >20,000 KJ (*n* = 1,360) (Meltzer, Brantsaeter, Ydersbond, Alexander, & Haugen, 2008). The MoBa Food Frequency Questionnaire (FFQ) in Q2 was not taken into use before March 2002, and before that, the women answered to a different FFQ, covering diet during the year prior to pregnancy, which explains the many pregnancies without dietary data. To avoid the use of multiple dependent observations, we included data from the first pregnancy for women participating in MoBa with more than one pregnancy, excluding *n* = 10,964 additional pregnancies by the same mother. In total, 71,648 mother–child pairs were considered eligible for the study. From these dyads, *n* = 17,873 had responded to the follow‐up questionnaire at 8 years. Infants with birth weight ±4SD (*n* = 526), participants lacking information on height and/or weight at 8 years were excluded (*n* = 2034) and additional *n* = 62 were excluded using a ±4SD approach when cleaning height, weight and BMI data. Children in the dataset who were <7 years (84 months) or >9.5 years (114 months) at the time of completing Q9 were also excluded (*n* = 262). The final sample used in this study thus included 14,989 mother and child pairs, which comprised 84% of the respondents with data from the 8‐year questionnaire and 21% of the mothers initially considered eligible for the study.

### 2.2 Main exposure

The primary exposure in this study was child adherence to the NND at 6 months, 18 months, 3 and 7 years of age assessed by previously developed child NND scores in MoBa (Agnihotri et al., 2020). These were developed with the intention to resemble the maternal NND score (Hillesund et al., 2014) and aimed to capture a potentially healthy and sustainable infant and child diet throughout childhood years. As the number of dietary variables in the child questionnaires was less specific and far less extensive than in the maternal FFQ, the developed child diet scores had to be adapted accordingly. Hence, the dietary components differ somewhat between the four child scores but is represented through their potential of contributing to a healthy, local or sustainable dietary pattern or dietary behaviours.

In brief, for each age, a variety of dietary variables from the questionnaires were selected to construct subscales as similar as possible to the subscales used in the maternal NND score. The mothers were mainly asked to respond to ‘How often does your child usually eat/drink the following’ with response alternatives varying slightly between questionnaires. All response options were recoded to reflect a weekly consumption. Missing was defined as having incomplete data on all food items that were included in construction of each child diet score. This was the case for *n* = 118 (0.8%) at 18 months, *n* = 461 (3.6%) at 3 years and *n* = 153 (1.2%) at 7 years within the final sample. For the remaining missing food items, an assumption of null intake was made in accordance with recommendations by Cade et al. (2002). These items were recoded to 0 (never/seldom) to avoid losing all dietary information for respondents with incomplete data for a given item. This was done for all scores, except for at age 6 months, as there were few missing items at this measure point. All respondents at each age assessment were included for development of the scores and in determining the cut‐offs (*n* = 89,315 at 6 months, *n* = 68,599 at 18 months, *n* = 57,911 at 3 years and *n* = 34,986 at 7 years). This was done to utilize most of the data and to get as representative cohort‐specific dietary information as possible. The cut‐offs of the subscales used to construct the diet scores were thus derived from a larger sample than the final sample used in this study.

The subscales were mostly dichotomized by the median (frequency of weekly consumption) and coded to give either 0 or 1 point, where receiving 1 point acknowledged a healthier food choice or a consumption above the median. Some subscales were scored according to responding ‘yes’ or ‘no’ to a question, where ‘yes’ indicated the favourable health behaviour. The sum of the subscales was further computed into a continuous NND child score. Finally, each score was divided into low, medium and high adherence groups with the intention to create as equally sized groups as possible. Where this was not possible, for example, where a certain cut‐off resulted in substantially larger low or high adherence groups, cut‐offs were chosen to yield the low and high adherence groups as equally proportioned as possible.

### 2.3 NND score at 6 months

The NND score at 6 months included homemade versus commercially prepared food, as reported by the mother, and breastfeeding as parts of potentially healthy and sustainable dietary habits. This score mainly captures the sustainability prospects of the NND and not the Nordic elements due to the limited number of diet variables in the questionnaire. The subscales of the score and the included food items of each score is presented below.

1. Consuming homemade fruit puree more frequently than commercially prepared fruit puree;
2. Consuming homemade dinners more frequently than commercially prepared dinners;
3. Consuming homemade porridge more frequently than commercially prepared porridge;
4. Being exclusively breast‐fed for at least 4 months;
5. Still being breastfed at the time of responding to the 6‐month questionnaire;
6. Drinking water far more frequently than sweetened beverages.

The subscales were summarized into a total score (0–6 points) and were divided into low (0–1 point), medium (2–3 points) and high (4–6 points) NND adherence at 6 months.

### 2.4 NND score at 18 months

The score at 18 months captures a potentially healthy diet and some prospects regarding locality/sustainability. The Nordic characteristics are mainly represented by potatoes, peas and beans, porridge and fish. The score at 18 months is briefly described as follows:

1. Fruits: eating fruits more than 10.5 times a week;
2. Vegetables: eating vegetables more than 5.5 times a week;
3. Peas and beans: eating peas and beans more than 5 times a week;
4. Potatoes: eating more potatoes relative to rice and pasta;
5. Porridge: eating more homemade porridge/baby cereal relative to commercially prepared porridge/baby cereal;
6. Fish: eating fish more than 2.13 times a week;
7. Milk: drinking more milk relative to fruit juice;
8. Water: drinking more water relative to sweetened beverages;
9. Homemade food: eating more homemade dinners relative to commercially prepared baby food.

After summarizing the subscales, the score values at 18 months ranged from 0 to 9 points and were further divided into low (0–3 point), medium (4–5 points) and high (6–9 points) NND adherence.

### 2.5 NND score at 3 years

The score at 3 years mainly represents a generally healthy diet, as more specific dietary variables related to the NND were not available in this questionnaire. The score at 3 years is briefly described as follows:

1. Fruits: eating fruits more than seven times a week;
2. Vegetables: eating vegetables or salad more than five times a week;
3. Potatoes: eating more potatoes relative to rice and pasta;
4. Fish: eating fish more than 2.12 times a week;
5. Milk: drinking more milk relative to fruit juice;
6. Sweetened beverages: drinking sweetened beverages less than 2.5 times a week.

The 3‐year score (0–6 points) was divided into low (0–1 point), medium (2–3 points) and high (4–6 points) NND adherence.

### 2.6 NND score at 7 years

The score at 7 years captures a Nordic diet more specifically than the former scores, as the dietary questionnaire was more extensive at this age. The score at 7 years is briefly described as follows:

1. Local fruits: eating apple, pear and grapes more than 3.5 times a week;
2. Root vegetables: eating carrots more than 1.5 times a week;
3. Cabbages: eating kale, cauliflower and broccoli more than 1.5 times a week;
4. Potatoes: eating more potatoes relative to rice and pasta;
5. Whole grain bread: reporting no consumption of white bread;
6. Oatmeal: eating muesli or oatmeal more than 1.5 times a week;
7. Fish: eating fish more than 2 times a week;
8. Milk: drinking more milk relative to fruit juice;
9. Water: drinking more water relative to sweetened beverages.

The sum of the subscales at 7 years of age yielded a score ranging from 0 to 9 points, and it was further divided into low (0–3 points), medium (4–5 points) and high (6–9 points) NND adherence.

### 2.7 Outcome variable

Child overweight was assessed from body mass index (BMI, kg/m^2^) computed from parent‐reported child height and weight at 8 years. The distribution of weight and BMI was skewed and was therefore logarithmically transformed before computing Z‐scores. BMI cut‐offs to assess overweight were age‐ and gender‐specific as recommended by Cole et al. (2000).

### 2.8 Covariates

Childhood overweight and obesity have been shown to be associated with many maternal and environmental factors (Trandafir & Temneanu, 2016). The following covariates were considered because of their potential association with the exposure and outcome: child sex, child birth weight (continuous), maternal educational attainment (12 years or less, 13–16 years, 17 years or more), maternal smoking during pregnancy (no/occasionally/daily), maternal age at delivery (years), parity (0–4 previous births), marital status (cohabitating/single), maternal prepregnant BMI from self‐reported height and weight (BMI < 25 kg/m^2^ vs. BMI ≥ 25 kg/m^2^) and maternal NND score. Child height, weight and BMI at 8 years (continuous) were included for descriptive purposes. Paternal BMI (BMI < 25 kg/m^2^ vs. ≥ 25 kg/m^2^) calculated from height and weight reported by the mother was included for the same purpose and for further examination of the data. Data on child sex, birth weight, maternal age, parity and marital status at delivery were derived from the MBRN, child height and weight at 8 years were retrieved from Q8, and the remaining information was obtained from Q1. Most of the maternal data was collected during pregnancy.

### 2.9 Statistical analysis

All statistical analyses were performed using the Statistical Package for the Social Sciences (IBM SPSS Statistics, version 24.0). Mother and child characteristics according to child NND adherence at 7 years are presented with proportions (%) for categorical variables and as means with standard deviations (SD) for continuous variables. Differences in means and proportions across the NND‐adherence categories were tested using Pearson's chi‐squared test and one‐way analysis of variance (ANOVA), respectively. To examine the association between the age‐specific NND scores and overweight at 8 years, we conducted separate binary logistic regression analyses with each of the NND scores, both in continuous and categorized form. This method was applied as the NND scores measured compliance to the NND differently at each time point across age, and a mixed effect model with the age‐specific NND scores as a repeated measure would not be a valid way of modelling trajectories at the individual level. With the continuous diet scores, we assessed the effect of a one‐point increase in diet score at each age, respectively, on odds of overweight at 8 years. In the categorized scores, low NND adherence was used as the reference group, and odds ratios (OR) for overweight for the medium and high adherence group were assessed with 95% confidence intervals (CI).

Crude and adjusted odds ratios were estimated in three models (crude, model A and model B), where the adjusted model (A) included child sex, maternal education, maternal age, parity, smoking during pregnancy, marital status, maternal prepregnant overweight and child birth weight. The last model (B) additionally included the maternal NND score, to remove a potential independent effect of maternal diet during pregnancy. Maternal prepregnant BMI and child sex was investigated for interaction with the relationship between diet scores and odds of overweight, but no evidence for an interaction was found (data not shown). Last, we performed a sensitivity analysis excluding preterm births (birth prior to 37 completed weeks) to assess potential influence on the findings.

### 2.10 Ethical considerations

The establishment of MoBa and initial data collection were based on a license from the Norwegian Data Protection Agency and the Regional Committee for Medical Research Ethics. The MoBa cohort is currently regulated by the Norwegian Health Registry Act. The current study was approved by The Regional Committees for Medical and Health Research Ethics (2019/339).

## 3 RESULTS

Child and maternal characteristics are described for the whole sample and according to low (29.2%), medium (43.4%) and high (27.4%) child NND score at 7 years of age (Table 1).

**TABLE 1:** Child and maternal characteristics of the sample according to level of New Nordic Diet‐adherence at 7 years

				Degree of NND adherence at 7 years (*N* = 12,704)						
		Whole sample		Low (0–3)		Medium (4–5)		High (6–9)		
		*N* = 14,989		*N* = 3,665 (28.8%)		*N* = 5,544 (43.6%)		*N* = 3,495 (27.5%)		
		Mean or *n*	SD or %	Mean or *n*	SD or %	Mean or *n*	SD or %	Mean or n	SD or %	*P* value
Child overweight at 8 years	No	12,855	85.8	3,152	86.0	4,762	85.9	3,000	85.8	0.925
	Yes	2,134	14.2	513	14.0	782	14.1	495	14.2	
Child BMI at 8 years (mean, kg/m^2^)		16.31	2.11	16.31	2.11	16.28	2.08	16.32	2.09	0.570
Child height at 8 years (mean, cm)		132.1	5.9	131.9	5.9	132.0	5.9	132.4	5.8	0.001
Child weight at 8 years (mean, kg)		28.6	5.0	28.5	5.0	28.5	5.0	28.7	4.9	0.085
Child birth weight (mean, g)		3639.5	525.7	3632.8	528.2	3639.9	522.8	3641.0	528.0	0.517
Child sex	Boy	7,541	50.3	1861	50.8	2,810	50.7	1738	49.7	0.463
	Girl	7,448	49.7	1804	49.2	2,734	49.3	1757	50.3	
NND score at 6 months, 0–6 (mean)		2.5	1.3	2.3	1.2	2.5	1.3	2.7	1.3	<0.001
NND score at 18 months, 0–9 (mean)		3.9	1.6	3.5	1.6	4.1	1.6	4.7	1.6	<0.001
Mean NND score at 3 years, 0–6 (mean)		2.7	1.4	2.1	1.2	2.7	1.3	3.4	1.3	<0.001
Mean NND score at 7 years, 0–9 (mean)		4.5	1.7	2.4	0.7	4.5	0.5	6.5	0.7	<0.001
Maternal NND score, 0–10 (mean)		4.8	2.0	4.2	2.0	4.8	1.9	5.6	2.0	<0.001
Maternal age at birth (mean)		30.4	4.4	30.3	4.3	30.4	4.3	30.7	4.3	0.001
Maternal educational level (completed years)	≤12 years	4,405	30.0	1,241	34.6	1,561	29.2	801	23.7	<0.001
	13–16 years	6,905	47.0	1,665	46.2	2,509	47.0	1,651	48.8	
	17 years or more	3,378	23.0	688	19.2	1,267	23.7	931	27.5	
Marital status	Cohabitating	14,602	97.4	3,569	97.4	5,412	97.6	3,418	97.8	0.443
	Single	387	2.6	96	2.6	132	2.4	77	2.2	
Parity	0	6,645	44.3	1,529	41.7	2,510	45.3	1,626	46.5	<0.001
	1	5,221	34.8	1,370	37.4	1917	34.6	1,129	32.3	
	2	2,451	16.4	610	16.6	885	16.0	560	16.0	
	≥3	672	4.5	156	4.3	232	4.1	180	5.2	
Smoking during pregnancy	No	13,797	92.5	3,324	91.2	5,149	93.2	3,296	95.0	<0.001
	Occasional	417	2.8	113	3.1	145	2.6	72	2.1	
	Daily	694	4.7	207	5.7	229	4.1	100	2.9	
Maternal prepregnant overweight (BMI > 25 kg/m^2^)	No	9,910	67.8	2,326	64.8	3,689	68.2	2,440	71.5	<0.001
	Yes	4,716	32.2	1,261	35.2	1721	31.8	973	28.5	
Paternal overweight (BMI > 25 kg/m^2^)	No	6,502	45.3	1,514	43.3	2,454	46.0	1,602	47.7	0.001
	Yes	7,852	54.7	1986	56.7	2,877	54.0	1757	52.3	

*Note*. Chi‐squared test for independence used for comparisons of categorical variables across adherence categories. One‐way ANOVA used for comparisons of continuous variables across adherence categories.

Abbreviations: NND, New Nordic Diet; SD, standard deviation; BMI, body mass index.

Children categorized with high NND score at 7 years of age had higher mean NND score at all previous time points compared to the lower NND categories. They were also taller than the children who were in the lower NND‐adherence categories. Mothers of children with high NND adherence at 7 years were more likely to have completed higher education, having fewer previous pregnancies and being older, and were less likely to be smoking during pregnancy or being overweight or obese pre‐pregnancy. There was no evidence for differences between the NND‐adherence groups regarding marital status, child sex, birth weight, child weight and BMI at 8 years or proportion with overweight at 8 years.

No differences in maternal NND score were observed between the mothers who were considered eligible for inclusion and the analysis sample (data not shown). There was, however, evidence for some differences in maternal and infant characteristics between the two groups. Compared with excluded mothers, mothers in the analysed sample were slightly younger, less likely to have smoked during pregnancy and more likely to have a prepregnant BMI ≥ 25 kg/m^2^. The analysed sample had a lower proportion of mothers with low and high educational level and a higher proportion with a medium educational level, and their offspring were slightly heavier at birth.

### 3.1 Regression analysis

Table 2 shows the association between a one‐point increase in age‐specific NND score and odds of overweight at 8 years. In the crude analysis, there was evidence for an association between NND‐score at 6 months and odds of being overweight at 8 years (Table 2, OR: 0.95; CI [0.91, 0.98] *p* < 0.003). This association was almost entirely attenuated in the adjusted model (OR: 0.99; CI [0.96, 1.03] *p* = 0.773). There was no evidence for an association between the individual NND scores and overweight at any of the other ages.

**TABLE 2:** Associations between a one‐point increase in the age‐specific NND‐scores and the odds of being overweight at 8 years

NND‐score (continuous)	*N*	Crude	*P* value	Adjusted	*P* value
		OR (95% CI)		OR (95% CI)	
Maternal score	14,258	1.00 (0.98, 1.03)	0.843	1.02 (0.99, 1.04)	0.195
6 months	14,206	0.95 (0.91, 0.98)	0.003	0.99 (0.96, 1.03)	0.773
18 months	13,057	0.99 (0.96, 1.02)	0.470	1.00 (0.97, 1.03)	0.916
3 years	11,774	0.97 (0.94, 1.01)	0.127	0.99 (0.96, 1.03)	0.775
7 years	12,007	0.99 (0.96, 1.03)	0.697	1.02 (0.99, 1.05)	0.236

*Note*. Adjusted for child sex, maternal education, smoking during pregnancy, maternal age at birth, parity, marital status, maternal pre‐pregnant overweight and child birth weight.

Abbreviations: NND, New Nordic Diet; OR, odds ratio; CI, confidence intervals.

In Table 3, the association between the categorical age‐specific NND‐scores and odds of being overweight at 8 years according to medium and high adherence with low adherers as a reference group is presented. In the crude model, children with high NND adherence at 6 months had lower odds of overweight at 8 years (OR: 0.81; CI [0.70, 0.94] *p* = 0.005) but not in the adjusted model. Further examination of the data showed that the inverse association between the NND score at 6 months and overweight at 8 years was mostly explained by maternal education and pre‐pregnant weight status (data not shown). No other evidence for an association between the age‐specific NND scores and overweight at 8 years was found.

**TABLE 3:** Associations between categorized age‐specific NND‐scores and odds of being overweight at 8 years

		Medium vs. low adherence	*P* value	High vs. low adherence	*P* value
		OR (95% CI)		OR (95% CI)	
Maternal score	(*N* = 14,258)				
	Crude	1.02 (0.90, 1.15)	0.797	0.99 (0.88, 1.11)	0.825
	Adjusted	1.07 (0.94, 1.21)	0.303	1.05 (0.93, 1.19)	0.408
6 months	(*N* = 14,091)				
	Crude	0.95 (0.85, 1.07)	0.401	0.81 (0.70, 0.94)	0.005
	Adjusted	1.05 (0.93, 1.16)	0.402	0.97 (0.83, 1.13)	0.724
18 months	(*N* = 13,057)				
	Crude	0.95 (0.85, 1.06)	0.345	0.94 (0.82, 1.08)	0.377
	Adjusted	0.96 (0.86, 1.08)	0.506	0.99 (0.86, 1.14)	0.883
3 years	(*N* = 11,774)				
	Crude	1.07 (0.94, 1.23)	0.303	0.94 (0.81, 1.09)	0.415
	Adjusted	1.10 (0.96, 1.26)	0.177	1.01 (0.87, 1.18)	0.887
7 years	(*N* = 12,007)				
	Crude	0.99 (0.88, 1.13)	0.935	1.00 (0.87, 1.15)	0.978
	Adjusted	1.05 (0.93, 1.20)	0.421	1.12 (0.97, 1.29)	0.117

*Note*. Adjusted for child sex, maternal education, smoking during pregnancy, maternal age at birth, parity, marital status, maternal pre‐pregnant overweight and child birth weight.

Abbreviations: NND, New Nordic Diet; OR, odds ratio; CI, confidence intervals.

We included the maternal NND score in a model B (model A + maternal NND) to remove a potential confounding effect of maternal diet during pregnancy on the odds of overweight at 8 years (data not shown). This did not change the estimates in either direction.

We performed sensitivity analyses by rerunning all models with preterm births excluded (*n* = 1,391, 9.3%). A noticeable change in estimate was only seen at 6 months with the categorical score at medium vs low (crude: OR: 0.98; CI [0.87, 1.12] *p* = 0.807, adjusted: OR: 1.09; CI [0.96, 1.24] *p* = 0.177) and high versus low (crude: OR: 0.84; CI [0.72, 0.98] *p* = 0.026, adjusted: OR: 1.01; CI [0.86, 1.19] *p* = 0.884).

Post hoc analyses including paternal weight status as a covariate in the model did not impact the results distinctively (data not shown).

## 4 DISCUSSION

Using data from a large prospective cohort study, we examined child NND adherence at 6 months, 18 months, 3 years and 7 years and their potential association with childhood overweight at 8 years. In unadjusted analyses, there was evidence for a protective association between higher diet score at 6 months (continuous and categorized) and overweight at 8 years; however, the observed associations were almost entirely removed after adjusting for potential confounders. The observed inverse association between NND score at 6 months and odds of overweight at 8 years in unadjusted results (Tables 2 and 3) was mostly explained by the educational level of the mother and maternal prepregnant weight status. Educational attainment is a commonly used indicator of socio‐economic position and may be associated with health and obesity through a variety of factors, such as income, occupation, health literacy and health behaviours (Cohen, Rai, Rehkopf, & Abrams, 2013). Possible mechanisms that have been proposed relating maternal prepregnant weight status to child adiposity are among others epigenetic effects of the foetal environment predisposing the foetus to later overweight/obesity (Lillycrop & Burdge, 2010). This emphasizes the importance of preventing overweight/obesity preconceptionally as excessive pre‐pregnant weight can affect the risk of overweight/obesity in future generations (Voerman et al., 2019).

Results from the performed sensitivity analyses suggest that the unadjusted inverse association between the diet score at 6 months and overweight at 8 years was influenced by the premature infants in the sample. This finding could represent a stronger protective association with breastfeeding and breastfeeding duration in this group of children or timing of complimentary feeding. Future studies are encouraged to examine this finding further.

Two of the diet score subscales at 6 months addressed breastfeeding, and some studies have shown that breastfeeding can be one of many factors that are protectively associated with childhood overweight/obesity (Lagström, Lande, & Thorsdottir, 2013; Spatz, 2014). The body of literature does, however, indicate that the protective effect is mostly evident during childhood and adolescence and that long‐term effects are unclear (Spatz, 2014). In a previous MoBa study, no evidence of a protective effect of full breastfeeding at 5 months, or breastfeeding beyond 1 year, was found on child weight development during the first 7 years of life, which is line with the findings in our study (Kristiansen et al., 2015). Another study has shown that breastfed infants provided with only homemade complementary food as compared to both homemade and commercially produced food had a significantly increased dietary diversity during the first year of life and lower levels of adiposity (Mok et al., 2017). As such, a combination of breastfeeding and serving exclusively homemade complementary food could be of particular benefit to the child's future weight status, although this could not be demonstrated in our data.

High NND adherers at 7 years had a higher mean NND score throughout childhood as seen from Table 1. Hence, an infant diet as captured by high NND score at 6 months could foster a healthier diet in later childhood, which has also been previously demonstrated in a MoBa study with other dietary indexes (Bjelland et al., 2013). Other studies have also confirmed a moderate tracking of dietary patterns from infancy to childhood (Lioret et al., 2015) and from early childhood to later childhood (Northstone & Emmett, 2008).

In a review of child diet quality indices (Marshall et al., 2014), only eight out of 24 studies reported an association between dietary patterns and overweight/obesity. The authors argue that this may partly be explained by poor study design and that data is derived from studies which are not specifically designed for the intended research question (Marshall et al., 2014). This could also be an explanation for the lack of evidence of an association in our study. We would expect a lower prevalence of overweight/obesity in the high NND group, as the mothers of these children had higher education, were less often overweight/obese and followed a healthier diet during pregnancy. Moreover, the prevalence of overweight 8‐year‐olds in our sample was 14.2%, which is lower than what was found in another Norwegian sample (20.2%; Glavin et al., 2014). We know that the mothers who initially chose to participate in the MoBa were older, more often cohabitating, nonsmokers and frequent users of multivitamin and folic acid supplement (Nilsen et al., 2009). This could suggest that there is an underlying possibility of self‐selection bias with a sample that is healthier than the general population and that child overweight is underrepresented (Nilsen et al., 2009). A potential association could have been attenuated due to this.

Furthermore, the children in the high NND‐adherence group were taller than the children in the middle and the low NND categories (Table 1). There were no differences in weight status across the categories, which may imply that adhering to a healthy Nordic diet can be related to growth in children. However, it could also be explained by a parental educational factor and thus a heritable component, as height has been associated with educational attainment (Galobardes et al., 2012). A strong educational gradient across the developed child diet scores has also previously been demonstrated in our data (Agnihotri et al., 2020).

We may not have been able to truly identify the overweight and obese children, as BMI as a tool may not be precise enough for such identification. Despite using age and sex‐specific BMI cut‐off values for overweight in children (Cole et al., 2000), it has been suggested that the scaling power of height in paediatric populations should be closer to 3 instead of 2, due to the developmental phase where weight is influenced by height to a greater extent (Cole, 1986; De Lorenzo et al., 2019). This was well demonstrated in U.K. birth cohort where low diet quality during early childhood was found to be associated with adiposity at 6 years of age but not with BMI (Okubo et al., 2015). As BMI failed to demonstrate an association where DXA was able to, the authors suggest that BMI may be an insufficient measure to identify adiposity in early childhood. An association between diet quality and objectively acquired weight status was also demonstrated in a cross‐sectional study of 1700 British children aged 9–10 years (Jennings, Welch, van Sluijs, Griffin, & Cassidy, 2011). Dietary quality was assessed using 4‐day food diaries and three predefined healthy diet scores. Anthropometric data were acquired by measurement, and the results showed that two of the scores but not the Mediterranean diet score (MD) were independently associated with weight status, even after adjustment for covariates known to be related to child overweight/obesity. The lack of an association between the MD and weight status was argued to be explained by the MD using the median to define adherence to the dietary pattern. As the sample followed a dietary pattern atypical to the MD, this may have resulted in some cut‐off points being uncharacteristic of the MD, contributing to an attenuation of a possible association (Jennings et al., 2011). Despite of being a widely applied method, using the median to assess cut‐off values has been largely debated, as it yields arbitrary cut‐offs that may result differently when used in different populations (Waijers, Feskens, & Ocke, 2007). This may also have been a factor of impact in our study.

### 4.1 Strengths and limitations

The data used in the current study derive from a large, prospective pregnancy‐cohort study conducted in Norway. The large sample size may be considered a strength, as well as the possibility to control for many potential confounders and assessing dietary data prospectively. However, there are limitations to our study. First and foremost, the developed child diet scores may not have been fully able to capture a true exposure to the NND, as the food variety in the child dietary assessment was limited. This has been discussed in detail elsewhere (Agnihotri et al., 2020). Although the diet scores aimed to identify a potentially healthy and sustainable diet, ascertaining sustainability is a complex venture (Burlingame & Dernini, 2012). Nonetheless, the Nordic diet has been regarded as an environmentally friendly diet (Meltzer et al., 2019; Ulaszewska, Luzzani, Pignatelli, & Capri, 2017) with health‐promoting prospects (Renzella et al., 2018).

Furthermore, most of the data were self‐reported. Possible social desirability and reporting bias cannot be ruled out, especially considering the sensitive nature of reporting body height/weight and possible undesirable food choices. It has been shown that energy intake of overweight and obese children is likely to be underreported, which may obscure potential associations between diet and the outcome of interest (Burrows, Martin, & Collins, 2010). Misreporting of the true consumption may also occur, as parents may have incomplete knowledge of what the child eats throughout the day, for example, in kindergarten, school or with caretakers who live separately. Measurement errors resulting in misclassification of diet score category is likely in this case and may have limited our ability to detect potential true associations between NND adherence and later overweight biasing the results towards the null.

We were not able to control for energy intake in the analyses. Higher scores in Nordic diet indexes have been associated not only with higher diet quality but also with higher energy intake and a slightly higher consumption of meat and non‐core foods, such as cakes and sweets (Bjørnarå et al., 2016; Hillesund et al., 2014; Roswall et al., 2015). Consequently, future scores should be energy adjusted to avoid potential confounding of associations by energy intake. However, as total energy intake is a suggested casual pathway to obesity, it has also been argued that such procedures may be an overadjustment, which in turn can lead to an under‐estimation of a true association (Ambrosini, 2014). Last, data collection at 8 years was still not complete in the applied data file, resulting in a smaller sample size which could have had an impact on the outcome in our study.

## 5 CONCLUSION

Children with high NND score at 7 years were on average taller, followed a pattern with a higher mean NND score throughout childhood, and were associated with a favourable socio‐economic position of the mothers, compared with the children in the lower categories. Application of NND scores to child diet at 6 months, 18 months, 3 years and 7 years showed no evidence of being protective against overweight at 8 years of age. Although this could reflect true null findings, methodological bias cannot be ruled out. Nonetheless, local and regional diets, such as the Nordic diet, are encouraged from both a sustainability perspective and for their other health‐promoting qualities and should be investigated in more detail in relation to other health outcomes.

## CONFLICTS OF INTEREST

The authors declare that they have no conflict of interest.

## CONTRIBUTIONS

ERH, NA, EB and NCØ contributed to the study conception and design. Data collection and data curation were managed by ALB. Material preparation and analysis were performed by NA, NCØ, AW, EB and ERH. The first draft of the manuscript was written by NA, and all authors commented on and revised following versions of the manuscript. All authors have read and accepted the final version of the manuscript.
